# WhatsApp-based intervention in urban Colombia to support the prevention of arboviral diseases: a feasibility study

**DOI:** 10.1080/20477724.2024.2358263

**Published:** 2024-05-24

**Authors:** Maria Angelica Carrillo, Alisa Maria Gessler, Tatiana Rivera Ramirez, Rocío Cárdenas Sanchez, Jörg Lindenmeier, Winfried V. Kern, Axel Kroeger

**Affiliations:** a Centre for Medicine and Society, Faculty of Medicine, University of Freiburg, Freiburg, Germany; b Research Group Geotecnia Ambiental (GIGA), University Francisco De Paula Santander, Cucuta, Colombia; c Graduate Institute, Geneva and Université de Lausanne, Lausanne, Switzerland; d Vector Control Programme, The Health Institute of Cucuta, Cucuta, Colombia; e Corporate Governance und Ethik, Albert-Ludwigs University Freiburg, Freiburg im Breisgau, Germany; f Center for Medicine, Universitätsklinikum Freiburg, Freiburg im Breisgau, Germany

**Keywords:** mHealth, mobile phone, WhatsApp, dengue, Zika, chikungunya, prevention

## Abstract

Arboviral diseases remain a significant health concern worldwide, with over half the world’s population at risk for dengue alone. Without a vaccine or targeted treatment, the most effective strategy of prevention is vector management with community involvement. mHealth interventions, like WhatsApp, offer promising results for engaging communities and promoting healthier behaviors. This study explores the feasibility of integrating WhatsApp in vector control activities to improve arbovirus prevention in Colombia. A mixed-methods approach was employed to assess the WhatsApp-based intervention. WhatsApp messages were sent to 45 community women for 5 weeks to increase their knowledge and practices about dengue, Zika, and chikungunya. Pre-and-post surveys and focus group discussions were conducted in community settings to measure the feasibility and acceptability of this intervention. Chat reviews were done to assess the usability of users. A total of 1566 messages were exchanged in 45 WhatsApp chats. High acceptance and good usability (82% of users used the app for replying) were reported in this study. WhatsApp messages were perceived as short, clear, and enjoyable. Users liked the frequency, and design of messages. Pre- and post-surveys demonstrated improvements in the knowledge and practices of arboviral diseases. The intention to apply this knowledge in practice was reflected in a significant improvement, particularly in cleaning the laundry tank once a week (pre 62.1% to post 89.6%, *p* < 0.008). This study suggests that using WhatsApp as an additional tool could be a feasible, acceptable, and affordable strategy for improving the adoption of better practices in the prevention of arboviral diseases.

## Introduction

Arboviruses are a diverse group of insect transmitted viruses that cause human diseases such as dengue, Zika, and chikungunya (DZC) which are all transmitted by *Aedes aegypti* and *Aedes albopictus* and are widely distributed in tropical and subtropical regions around the world [1]. These three arboviral diseases are devastating with over half of the world’s population at risk. Dengue is one of the most rapidly increasing vector-borne diseases, and results in 100 million cases reported annually [2]. Chikungunya and Zika have also caused large epidemics in the Americas and elsewhere that rapidly spread through a large, susceptible population [3]. Vector management is currently the primary available prevention strategy as the first dengue vaccine still has limitation in its use and several others are under development [4]. The most common vector control method in many countries is the application of the larvicide temephos to domestic water containers or space spraying with an insecticide for adult mosquitoes, followed or combined with biological control. However, the limited success of these methods can be attributed to increasing insecticide resistance [5,6], as well as to cultural and social factors related to living conditions and to behaviors and practices of the population that favor the proliferation of *Aedes* spp. breeding [7].

Initiatives such as integrated vector management (IVM) have been promoted to tackle these issues through a functional integration model to reduce vector densities and ultimately disease incidence [8]. In line with this approach, there have been programs that involve community mobilization and behavioral change for improved dengue prevention and control. However, the difficulty of these programs lies in finding ways to achieve the active and sustainable participation of local people. Well-constructed social strategies such as Communication for Behavioral Impact (COMBI [9]) that incorporate information and communication technologies (ICTs) could play a role in increasing community engagement of potential risks and encouraging collective action but have not led to a sustainable vector control.

During the last decade, mobile-based interventions have received considerable attention to improve population access to health information and care [10,11]. This approach articulated with health education can be beneficial for improving health outcomes [12,13]. Research in Peru and Nepal has demonstrated that delivering health messages through Short Message Services (SMS) can promote positive changes in human behavior, improving dengue practices and consequently decrease vector densities in households [14,15]. The use of mobile devices in the health field is referred to as mobile health (mHealth) [16]. Since the arrival of advanced mobile devices called ‘smartphones’, there has been a special focus on integrating or creating mobile applications (apps) for a wide range of health-related needs [17], including arboviral diseases. Novel mobile applications have been developed for different purposes in the prevention and control of DZC. To date, these technologies are mainly aimed at the collection and reporting of cases and symptoms, monitoring outbreaks, tracking mosquitoes, and mapping breeding sites and hotspots [18]. However, only few studies have explored the use of mobile phones in health education and promoting behavior change.

WhatsApp Messenger (WhatsApp Inc.) is one of the most popular mobile applications worldwide, with approximately 2 billion active users [19]. Although its primary purpose is social networking, the popularity of WhatsApp has meant that it has been widely used as a channel for sharing news and information [20]. This feature has been leveraged for health systems and programs for improving communication between patients and professionals or health authorities and communities, favoring the spread of health-related information [21], creating reliable communication channels and promoting healthier behavior in different target populations [22,23]. Despite these benefits, no research has explored the use of the WhatsApp application in the prevention of dengue, Zika, and chikungunya yet.

In Colombia, there is increasing interest to implement technological solutions in vector control operations such as development of software for epidemiological analysis, mosquito tracking [24,25] and mobile applications for data collection [26]. However, these initiatives are only focused on vector surveillance rather than improved education or community engagement. In 2019, a national survey by DANE (National Statistics Department) reported a massive increase in ownership of mobile phones. Approximately 95% of households have at least one mobile phone and around 80% of mobile phone users have a smartphone [27]. Additionally, a recent digital report showed that a high proportion of Colombian users (95.6%) use their phone to access internet and 41% check their health symptoms online, which is higher than the worldwide average (25%) [28]. WhatsApp is the most downloaded messaging app among mobile users in Colombia [29]. Moreover, a recent study shows that instant messaging and social networks are the communication methods preferred by Colombians [30].

Researchers from Freiburg University in Germany (Center for Medicine and Society, ZMG) and from the State Health Institute of Norte de Santander (IDS) in Colombia tested a novel vector control method, ‘insecticidal coating for water tanks’, as an alternative to controlling *Aedes* spp. mosquitoes in Cucuta and its two adjacent municipalities in Colombia. These areas share the main land border crossing with Venezuela, alongside certain unauthorized points of entry. Over the past seven years, forced displacement and migration waves have led to over 2.8 million migrants and refugees fleeing from Venezuela to Colombia [31], with around 337,000 of them staying in the State of North Santander [32]. These populations are also at risk and could benefit from the insecticidal coating. The coating is applied in water tanks for laundry which are the main mosquito breeding places in the city. Safety assessments and long-term effectiveness of the method were proved in scientific papers [33–35]. This vector control method aims at involving local communities as much as possible as active partners who ensure the acceptance of the measures, support the promotion and participate as community volunteers in the painting of water containers in their neighborhoods. In the communication between vector control staff and community, digital devices such as smartphones could have a major role. Additionally, a sub-survey was conducted in 2000 households in Villa del Rosario and Los Patios as part of the baseline study to assess the transmission risk of arboviral diseases and people’s willingness to apply insecticidal coating [36]. It was found that 91.7% of the population had smartphones with WhatsApp, and 81.1% of individuals were interested in receiving preventive messages on their mobile phones. Given these facts and the possibility to introduce WhatsApp as part of vector prevention efforts, this study aimed to test the feasibility of a digital (mHealth) intervention to provide health education and social engagement for community women living in the metropolitan area of Cucuta, Colombia. The intervention focused on the prevention of dengue, Zika and chikungunya considering the following areas: knowledge of arboviral diseases, preventive practices, promotion of novel vector control tools and other relevant health information.

## Methods

The study aimed to explore whether a WhatsApp-based intervention for community women can improve the knowledge and practices related to arboviral disease prevention. Further, the study aimed to validate and assess the acceptability of using WhatsApp messages as a service for health promotion targeting communities affected by dengue, Zika and chikungunya.

### Setting

This study was part of a large-scale project on the effectiveness of tank-coating for reducing dengue incidence in pre-selected neighborhoods with the highest rates of DZC in two urban municipalities (Villa del Rosario and Los Patios) within the metropolitan area of Cucuta, the capital of Norte de Santander, Colombia [36]. Villa del Rosario is located at an elevation of 440 m and covers an area of 228 km^2^, whereas Los Patios is 410 m high and has a spread of 131 km^2^. Both areas are close to the Venezuelan border and heavily affected by migration. The registered population is 92,661 inhabitants in Villa del Rosario and 78,409 in Los Patios. The average annual temperature of 27°C provides ideal conditions for the breeding and spread of *Aedes* spp. mosquitoes, the vectors of DZC [37].

In relation to the latest distribution of dengue reported by the National Health Institute, the incidence rate in Cucuta stood at 229.3 per 100,000 individuals in 2021, which was higher than the national rate of 165.0 per 100,000 [38]. Villa del Rosario and Los Patios had dengue incidence rates of 196.3 and 197.5, respectively. However, these figures were estimated during the COVID-19 crisis. Prior to the pandemic, the average annual dengue incidence rate in Cucuta was alarmingly high at 400 cases per 100,000 between 2009 and 2019 [38]. Recently, a large study conducted in Cucuta, Villa del Rosario, and Los Patios estimated a high vector infestation of 25.1% in the overall house index, which was above the 5% assumed by WHO, indicating a high risk of epidemic transmission [36]. Traditionally, these communities have used laundry tanks, known as ‘lavaderos’, for washing clothes and cleaning houses. This type of container was reported as the most productive breeding site for *Aedes* spp. mosquitoes, as they are frequently uncovered and situated outside of the house [36].

### Study design

The current study used an explanatory sequential mixed method design following the guidelines by WHO [39] to evaluate a WhatsApp-based intervention as part of the above-mentioned implementation trial for dengue vector control. A pre-and-post design without a control group was conducted to measure the change in knowledge and practices of community women during August – October 2019. After the follow up survey, two focus group discussions were conducted to validate the structure and content of messages and assess the perceptions of the WhatsApp intervention. Furthermore, the usability was also measured through chat reviews. Recommendations were provided for the adoption of the WhatsApp message intervention. During COVID-19, all implementation trial activities had to be stopped. Nevertheless, communication with community members was maintained. After this period, formal monitoring of the mHealth intervention restarted and compliance was followed informally until July 2023. Results and analysis of the first phase before the interruption are included in the present study.

### Study participants and recruitment

**Figure 1.**
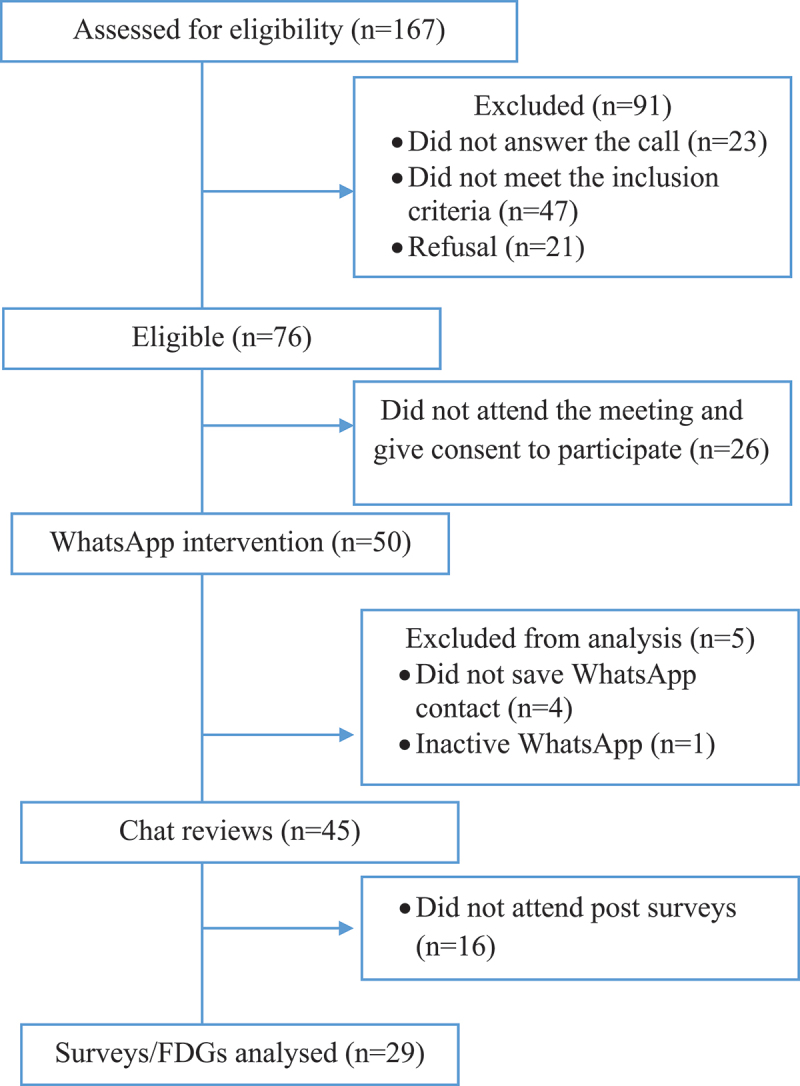
Flow of participants throughout the study.

Participants were community women aged 18 years and above, from areas of Villa del Rosario and Los Patios. The involvement of women can be effective in vector control programs at the community level [40]. Women are usually more active in reduction of mosquito breeding sites because they are often responsible for household water management and family care, particularly in low-and-middle-income countries [41,42]. Engaging a group of women in the identification and elimination of the most productive containers has shown to be successful in reducing mosquito abundance [43,44]. Therefore, this study contacted women associations with National Colombian programs such as Families in action and Family, Woman and Childhood. These associations identified and contacted women leaders to be recruited in this study. Women were eligible for this feasibility study if they had (i) a smartphone with good internet connection, (ii) experience using WhatsApp, and (iii) lived in the study areas. Eligible women were invited to a meeting to explain further details of the study. After providing their verbal and written informed consent, the participants were selected and registered in the WhatsApp application. The selection process of participants was developed in four steps (see Figure 1). We ended up recruiting 50 participants for sending WhatsApp messages, but the study had a loss-to-follow up (*n* = 21) which resulted in 29 participants being included in the survey/FDGs analysis. Furthermore, this study examined the recorded chats of all participants (*n* = 45) to assess the application’s usability.

The flow diagram shows the selection process of the participants throughout the study.

### WhatsApp messages design and development

WhatsApp is an existing application worldwide which was implemented during this study as a mHealth app for health education, health promotion, behavior modification and health communication of arboviral diseases. WhatsApp (version 2.19.241) for Android device was used since all participants had this operating system in their smartphones. Reminder messages were sent through the WhatsApp broadcast function using a simple smartphone model (Moto G). The project name was mobile health because the primary purpose was to disseminate health information with emphasis on arboviral diseases.

**Figure 2.**
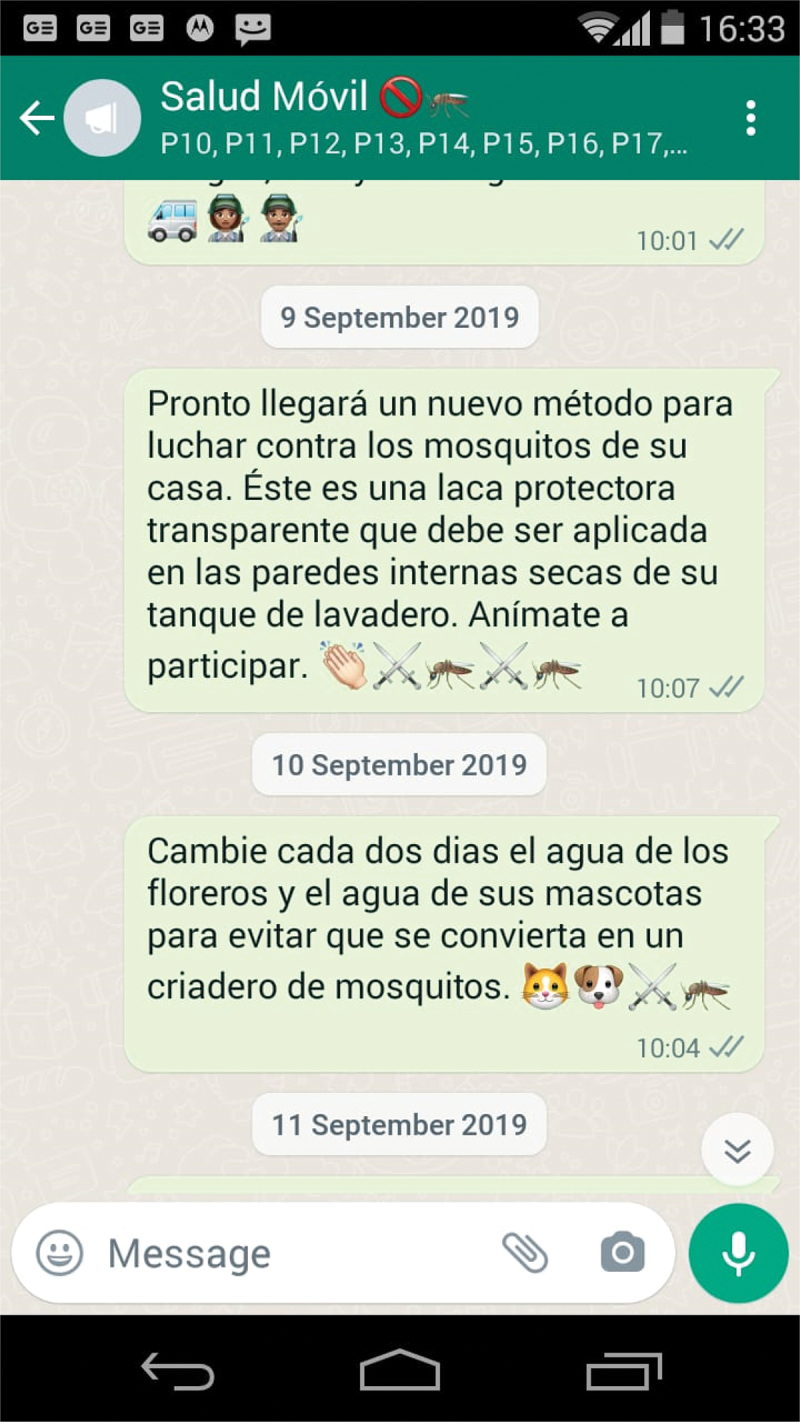
WhatsApp messaging.

All WhatsApp messages were developed following the national guidelines for the prevention and control of DZC [45] and recommendations of previous studies on mobile technology for dengue [18]. In total, 20 short text messages (no more than 350 characters each) were designed for this study. Two additional text messages were incorporated to welcome and thank for the participation in this study as well as one voice message was included for Valentine’s Day in Colombia. Emojis were included in each text messages for making them more readable, lively and user-friendly. An example of WhatsApp messaging is presented in Figure 2. All messages were written in Spanish language and reviewed by local health staff and national and international experts in arboviral diseases. Messages are described in the additional file 1.

Figure legend 2: Figure 2 presents a screenshot of the WhatsApp messaging using the broadcast function which was captured by the facilitator (researcher AMG) who sent the messages from the project’s mobile phone.

Messages have four components: (1) health education, (2) health promotion (3) behavior modification, and (4) health communication. These components aimed to improve knowledge, promote a new novel vector control tool for water containers, increase intention-to-change behavior on preventive practices against arboviral diseases and disseminate relevant health information.

### Health education

The WhatsApp intervention contained 5 messages with educational information on the three target diseases. Vector and its habitat (breeding sites), warning signs of DZC, and transmission patterns of arboviral diseases were included in this component and then validated by national and international health experts and adjusted by local health staff. Example of message: ‘A breeding site is any water reservoir inside and outside the house where the *Aedes* mosquito lays its eggs, which develop to larvae and eventually into new mosquitoes that can transmit dengue, Zika and chikungunya’.

### Behaviour modification

Eight messages on how to prevent DZC with emphasis on the protection of domestic water containers were developed based on the Communication for Behavioural Impact (COMBI) tool. This approach employs behavioral models as well as communication and marketing theories to engage individual and families in the acquisition of recommended healthy behaviors and encourage the adoption of those behaviors toward the prevention of dengue [9]. Messages were designed as reminder, but also some of them were formulated as questions or instruction for encouraging users to respond through any type of communication (e.g. photo) when they participated in a preventative practice. Some examples were: i) ‘I hope you have not forgotten to clean your laundry tank, especially the walls, corners and joints of your tank’, ii) ‘Did you change the water in the flowerpots and/or the water of your pets’ bowls?’.

### Health promotion

Five messages with promotional information on the new vector control method (insecticidal coating) and how to use it were designed following the instructions of insecticidal coatings which were developed by Inesfly Corporation (manufacturer of the insecticidal coating) and tested by the authors [35,36]. e.g.: ‘A new method to combat mosquitoes in your home will soon arrive. This is a transparent protective coating that should be applied to the dry walls of your laundry tank. Let’s participate!’.

### Health communication

The purpose of this component was to provide general information on the national and regional health service. One message was illustrated with the slogan of the current national campaign against dengue ‘Cut the wings of dengue’ developed by Ministry of Health (MoH) [45] and a text message related to health staff. e.g.: ‘Allow the staff from the Departmental Health Institute enter your home and follow the instructions related to *Aedes* mosquito control to prevent dengue, Zika, and chikungunya’.

### WhatsApp intervention

All women who had signed up for participation in the WhatsApp trial received a private welcome message, reminding them of saving the phone number. The WhatsApp broadcast function was used to send messages to each participant which allowed easy distribution and anonymity protection in comparison with WhatsApp groups. Participants did not have access to information from others. It was necessary for each participant to save the project number in their own mobile phone as a condition for receiving broadcast messages via WhatsApp, according to the regulations of the app. The WhatsApp infographic function served as a process indicator to monitor message reception, which was previously accepted by the participants through informed consent.

WhatsApp messages were sent anonymously to each registered WhatsApp chat by the author of the study (facilitator). The facilitator was responsible for managing the WhatsApp application and interacting with participants (Table 1). The facilitator responded to messages during working hours, ensuring a timely response within one day. They had the freedom to ask questions if they had any doubts about the intervention or were interested in topics related to the management of arboviral diseases and the use of insecticidal coating Messages were sent at 10 a.m. every day (excluding weekends) which was previously agreed upon by everyone. Participants were allowed to respond to messages using text messages, voice messages, videos, and any other reactions since this WhatsApp intervention was designed for a bidirectional communication. In the pre-standardized messages, three were designed to prompt behavior change by asking questions like ‘Did you already covered or turned around all buckets, pots, and bottles in your house?’. It was not mandatory for participants to interact or respond to each message. Participants could choose to withdraw from the study at any time. Participants were also instructed not to forward these messages to anyone until the end of the study. The period of intervention was 23 workdays (approximately 5 weeks).

**Table 1.** Role of the facilitator.

Primary responsibilities of the facilitator in the study
(1) **Sending Daily Messages**
Sending pre-designed messages to participants during workdays and ensuring messages are sent according to the predetermined schedule.
(2) **Addressing Participant Inquiries**
Responding to inquiries from participants regarding the prevention and control of arboviral diseases, the insecticidal coating, and any information about the study.
(3) **Providing Technical Assistance**
Assisting participants in navigating any technical challenge they encounter with the WhatsApp application.
(4) **Reporting Technical Issues**
Identifying and reporting technical issues or glitches with the WhatsApp application promptly.

### Research instruments

Pre-and-post-intervention surveys were designed and adjusted following questions on Knowledge, Attitudes, and Practices (KAP) of dengue in Colombia [46]. They were validated by experts and local health staff through participatory workshops. Pre- and post-surveys were disseminated in person during two meetings, the first one for registration in the study and the second one for feedback and closure of the study. The pre-survey had five sections: sociodemographic information, knowledge, practices data and willingness to participate in vector control activities. The post-survey had the same sections with additional questions related to the novel vector control tool and acceptability of the intervention. Extracted information from surveys is found in the additional file 2.

Immediately after the post survey, all women were invited to participate in focus group discussions (FGDs) which were conducted by three female interviewers (AMG, MAC, RCS) who were trained to conduct qualitative research. A total of 29 women participated in two FGDs. They were designed to gather information on women’s perceptions and acceptability of the WhatsApp-based intervention and to validate the WhatsApp messages. The FGDs included five themes on women’s experience with the messages, users’ satisfaction, content of the messages, time and frequency of the messages, and suggestions to improve the WhatsApp program. Both FGDs lasted between 40 and 55 min. The discussions were audio-recorded and transcribed for analysis. Examples of questions were: How did you feel using WhatsApp for dengue prevention? What was your favorite message? How do you think these messages could help you increase DZC prevention?

The WhatsApp chat reviews were collected using the export chat function from the same application to obtain text script of each chat. Text scripts received as input the raw transcript of all messages by the facilitator, participants, and content of each message. Scripts were transferred into a database of the Excel program to evaluate user’s interaction and usability of the intervention. Furthermore, scripts were also extracted and categorized by type of communication of users (text, voice, emoji, among others), usage (frequency of messages by the facilitator and users) and type of user (active or inactive). Extracted chats are found in additional file 3.

### Statistical analysis

The quantitative data collected before and after the intervention study were analyzed using the Statistical Package for the Social Sciences (SPSS) version IBM SPSS Statistics 29.0.1.0. The levels of participants’ arbovirus knowledge and practice were scored. They were the primary outcome indicators of the study. The score attributed to each question ranged from 0 to 1, with a maximum score of 29 points as the total number of participants was 29. Therefore, only 1 point was marked for the correct answer and 0 points were marked for the wrong answer. The differences between pre and post scores were tested using the McNemar test. A p-value <0.05 was considered statistically significant.

The qualitative data were analyzed by themes. After reading all FGD transcripts, the research team identified patterns and used inductive coding to identify keywords and concepts to develop a list of common codes to analyze the data. To ensure the reliability of the analysis, a qualified qualitative researcher independently coded the de-identified transcripts. The two coding schemes were then reviewed, discussed, and developed into the final coding scheme by the research team. A total of five themes were analyzed using the grounded theory approach for the qualitative analysis [47]. Additionally, FGDs were conducted followed the guidelines by WHO [48]. The text was extracted from transcriptions to generate analysis content, themes, and findings, and then translated to English.

Chat reviews were calculated with the total number of messages per frequency of messages sent by women and the facilitator. Each interaction of women was also evaluated to estimate the usability in terms of active users who read the WhatsApp messages and users who used the app at least replying once (any type of communication was accepted as a reply).

## Results

Fifty women were registered in the WhatsApp messaging application to receive the mHealth intervention for 5 weeks, but only 45 of 50 women received the WhatsApp broadcast messages. The main challenges were failure of saving the WhatsApp project number on cell phones and having an inactive WhatsApp connection. According to the infographic function, 4 of the 50 initial participants did not save the number and 1 participant had her WhatsApp offline, thus they were unable to receive all messages until follow-up. After the intervention, 29 women completed the evaluation questionnaire and participated in the FGD (see above Figure 1).

### Sociodemographic characteristics of the study population

The baseline characteristics of the 29 women are described in Table 2. Most community women lived in Villa del Rosario (62.1%), had a mean age of 40 years (range 20–68 year, SD 12.09), had completed secondary studies (44.8%) and were housewives (44.8%). None of them reached a university degree.

**Table 2.** Sociodemographic characteristic of study population.

Characteristic	Variable	% (n)*
Setting	Villa del Rosario	62.1% (18)
	Los Patios	37.9% (11)
Age group	<30 years	13.8% (4)
	30–49 years	62.1% (18)
	>49 years	24.1% (7)
Educational level	Primary education	31.0% (9)
	Secondary education	44.8% (13)
	Vocational education	20.7% (6)
	No education	3.4% (1)
Occupation	Housewife	44.8% (13)
	Educational agent	17.2% (5)
	Hairdresser/manicurist	10.3% (3)
	Public employed	3.4% (1)
	Merchant	3.4% (1)
	Other occupations	10.3% (3)
	No response	10.3% (3)

*The universe for this study comprised the 29 participants who were included for the analysis.

### Knowledge of arboviral diseases

Table 3 shows a considerable improvement of knowledge levels of women about arboviruses-transmitting mosquitos (mosquito’s name: 58.6% to 89.7; *p* < 0.004; mosquito’s gender: 55.2% to 82.8%; *p* < 0.008) and the water sources for the development of *Aedes* spp. vectors which improved by 27.6% (*p* < 0.021). The post-intervention knowledge of women about breeding sites improved by 17.3% but it was insignificant regarding the warming signs of DZC (*p* < 0.180). Participants were familiar with the symptoms of dengue and have likely heard about how to prevent it because they came from dengue-affected areas where prevention campaigns have been conducted before. The three most frequently mentioned symptoms were fever (93.1%; *n* = 27/29), headache (58.6%; *n* = 17/29), and skin rashes (31,0%; *n* = 9/29). After the WhatsApp intervention, all the women (*n* = 29) felt well informed about the transmission and prevention of DZC.

**Table 3.** Knowledge of community women about DZC.

Knowledge topics	Pre-intervention % (n)	Post-intervention % (n)	P-value
DZC-causing *Aedes* spp. Mosquitoes	58.6% (17)	89.7% (26)	0.004
Transmission by female *Aedes* spp. mosquitos	55.2% (16)	82.8% (24)	0.008
Breeding sites for *Aedes* spp. mosquitoes	79.3% (23)	96.6% (28)	0.063
Water source for breeding/development	62.1% (18)	89.7% (26)	0.021
Three or more DZC symptoms	72.4% (21)	89.7% (26)	0.180

### Practices about the prevention of DZC

Table 4 presents the proportion of women who were aware of the need to take action regarding two specific activities for preventing DZC transmission. Before the intervention, a high proportion of women knew about the recommended frequency of changing water in pet bowls (93.1%; *n* = 27) and cleaning laundry tanks (82.8%; *n* = 24). However, in practice, only around 62% of them followed these recommendations. After the intervention, there was a significant improvement in the adoption of DZC prevention measures. The number of women (excluding 8 who had no pets) who changed water of their pets’ bowls at least every 2 days increased to 33.3% (*p* < 0.016), and those who cleaned their laundry tanks at least once a week rose by 27.5% (*p* < 0.008). These findings indicate a positive change in the DZC preventative practices in community women.

**Table 4.** Practices for the prevention of DZC in women.

Activities for the prevention	Knowledge and practice	Pre-intervention % (n/N)	Post-intervention % (n/N)	P-value
Changing the water in pet’s bowl (CWP)	Knowledge on CWP at least 2 days	93.1% (27/29)	96.6% (28/29)	1.000
	Practice CWP at least 2 days	61.9% (13/21)	95.2% (20/21)	0.016
Cleaning of laundry tank (CLT)	Knowledge on CLT at least 1 week	82.8% (24/29)	89.6% (26/29)	0.625
	Practice CLT at least 1 week	62.1% (18/29)	89.6% (26/29)	0.008

### Acceptance of WhatsApp intervention

The WhatsApp intervention for improving knowledge and – more importantly – action for the prevention of DZC was well accepted by the 29 women participating until the last phase (participating in FGDs). All of them responded that WhatsApp is an excellent tool for disseminating information on arboviral diseases and improving the willingness to participate in vector control operations, particularly in the application of insecticidal coating for water containers.

From FGDs, women perceived that receiving WhatsApp messages for DZC prevention were a good, useful, and motivating strategy. They agreed that the messages could be used as a source for prevention of arboviral diseases.

- ‘It seems very good to me, the idea of WhatsApp messages because that way one can communicate it to the family and it is something that one can do at home, pick up things, clean the jars, but one doesn’t know that there is a good strategy to eliminate the mosquito’ (Participant 11, VdR)
- ‘The messages helped us, for example in my home they helped me a lot to prevent, to be more vigilant about the things that … avoid breeding sites and avoid mosquitoes breeding and also avoid a dengue or Zika infection’ (Participant 4, LP)
- ‘The messages were very motivating, very excellent, very clear and they helped to reinforce that… this is commitment that one has’ (Participant 1, LP)

Reminder messages via WhatsApp were the favorite channel to send health information in comparison with alternatives media such as flyers and Short Messages Services (SMS).

- ‘One never reads them [flyers], instead the messages, it’s like you’re so connected to reality when you read one every day. That’s the way it is. But this flyer, one never reads it’ (Participant 2, VdR)
- ‘SMS are boring’ (Participant 4, LP)
- ‘I do not read text messages via SMS because there are a lot of advertising for other things, I trust WhatsApp for the health promotion’ (Participant 13, VdR)

Some women also mentioned that the messages help them to increase awareness and adopt a better behavior concerning the elimination of *Aedes* spp. mosquitoes.

- ‘Be aware, awareness. To be aware there is an enemy and it is in the house’ (Participant 3, VdR)

Even, some of the women sent pictures through WhatsApp doing preventative activities, particularly washing tanks which were also registered in the chat reviews.

- ‘I even took a picture washing the laundry tank … so that you see that we are attentive to the project because it is very good and beneficial for everyone’ (Participant 18, VdR)

However, some practices were not followed due to technical problems with WhatsApp messaging, the lack of water in some communities or some participants were negligent.

- ‘This last [message] did not reach me’ (Participant 2, LP)
- ‘I changed my number’ (participant 11, LP)
- ‘I forgot it’ (participant 5, LP)
- ‘Since I didn’t get water, I emptied the tank and I already had little water’ (Participant 11, VdR)
- ‘I don’t get water every day, and I’m not going to throw it away’ (Participant 17, VdR)

Some women suggested that they could be part of this project as ‘multipliers’ and willing to sensitize their communities.

- “‘Let’s all share on social media and more that we start to be part of a project, yes! I talk to my neighbor, my family, and so on. What we want is to improve. Well, like us to spread the word, like us to be multipliers and carry out this awareness’ (Participant 1, LP)

Furthermore, the use of WhatsApp was recommended for other health projects, beyond the insecticidal coating.

### Content of WhatsApp messages

During the intervention period, a total of 1,566 messages were exchanged in 45 WhatsApp chats over 5 weeks, of which 72% (*n* = 1132) were sent by a facilitator and 28% (*n* = 438) were sent by women participants. A list of intervention messages sent by the facilitator is displayed in Supplementary Additional file 1. Emoji were the main type of communication used by women (51.6%) to express their satisfaction or agreement or compliance, followed by text messages (27.6%) (Table 5). Since the intervention messages were the same across the chats, analyses are presented with all WhatsApp chats combined.

**Table 5.** Type of response message sent by community women to the facilitators.

Type of message	% (n)
Message with only Emoji	51.6% (226)
Text message	27.6% (121)
Text message with emoji	5.5% (24)
Voice message	9.4% (41)
Picture	3.6% (16)
Video	0.5% (2)
Gift	0.2% (1)
Sticker	0.2% (1)
Link/contact shared	0.5% (2)
Deleted message	0.9% (4)
Total messages	438

Each message was read and discussed by participants during the FDGs. The content of messages was short, clear, and enjoyable. Generally, women liked the time (10 am), frequency, size, and design of messages, in particular the emojis in the end of every message.

- ‘Something that is important is that the messages are short so that people are going to read it quickly’ (Participant 11, VdR)

- ‘It’s short!, It’s very clear’ (Participant 8, LP)

- “Yes at 10, Yes [every day]. We all want you to write to us (Participant 13, VdR)

- ‘All the messages were very enjoyable, even my son liked the messages especially the emojis at the end. they and even their children liked the messages especially the emojis at the end’ (Participant 6, VdR)

No one reported difficulty when using the app or asked for training on how to use it because they were familiar with the use of this digital tool. However, some words were suggested to be corrected such as ‘Infectious disease’ is preferred over ‘contagious’ and the ‘elevated tank’ should better be called ‘aerial tank’.

### Usability

From the fifty women registered in this study, 45 women (90.0%) received WhatsApp messages. Among these active users, 39 (86.6%) of them were able to read all messages as verified by infographic function. Some technical issues meant that a few women did not manage to read all the messages because they initially did not save the project number (which made it impossible to deliver messages by broadcasting function), intermittent internet, and changing phone numbers.

In terms of response, it was found that 37 of active users (82.2%) used the app for replying at least one message and 18 of them (40%) were actively involved replying more than 10 messages and 7 users sharing a photo when they did wash their water container. Only 8 (17.7%) participants did not respond messages even once, most of them coming from Los Patios (*n* = 7). The messages that received the most interaction were those whose purpose was to promote insecticidal coating and preventive practices toward dengue (more than 30 replies). The messages that received the least interaction were those related to health communication (only 3 replies). No difference was observed between the frequency of days of the week they used the app (average response messages per day = 9 replies).

### Costs of WhatsApp intervention

Financial costs are presented according to three major cost items (personnel, material, and technology), and program period (preparation and implementation) (Table 6). Over the 5-week period, the total financial costs were US$876 (U.S. dollars); the main cost item was staff cost (facilitator). This amount may increase if the number of users increases as it could require personnel with IT skills, more technological capabilities (computer and software), and licensing permits of WhatsApp providers and service.

**Table 6.** Cost of intervention over 5 weeks.

Type of costs	Costs (COP $)	Cost (US $)*
**Preparation costs**		
Meeting for presentation and training	500,000	151
Smartphone	600,000	181
Materials, and printing (pre surveys)	150,000	45
Total	1,250,000	377
**Implementation costs**		
Mobile phone basic plan (55G over 5 weeks)	74,000	22
Facilitator (over 5 weeks)**	1,430,679	432
Materials and printing (post survey)	150,000	45
Total	1,654,679	499

*1US 1$= COP $3,313 on 1 August 2019.

**Based on the minimum wage, one day’s work is equivalent to $38,667 COP.

## Discussion

This is the first study to use the WhatsApp application as a health digital tool for supporting the prevention of arboviral diseases. The study introduced and described the design and development of text messages for a mobile application and the testing in urban communities of Colombia. One of the major findings was demonstrating that the incorporation of mHealth-based interventions through an existing and well-known application such as WhatsApp in vector control programs is feasible and acceptable for certain population groups, particularly women. Previous studies have also demonstrated that the WhatsApp-based intervention is feasible for improving health outcomes in women [49,50].

### Improving knowledge and practices

Although from the beginning, participants showed prior knowledge of certain prevention measures to reduce mosquito breeding, there was a significant increase in the application of this knowledge, particularly in actions associated with water containers. After the intervention, there was a clear trend toward the improvements of preventive practices, especially cleaning the laundry tank once a week (pre 62.1% to post 89.6%, *p* < 0.008). These results may suggest that WhatsApp may not only increase knowledge, but also improve preventive practices. This result is similar to other implementation studies conducted in Peru and Nepal that show improvements in knowledge and positive changes in the practices toward the dengue prevention when participants are exposed to health information through SMS messages [14,15]. However, the integration of communication technologies such as mobile applications or SMS into vector control programs should depend on local needs, technological capabilities, and preferences of the population [18]. Further studies should be conducted at scale to reconfirm this finding.

### Acceptability, usability and cost

The acceptance of the WhatsApp messaging for the prevention of arboviral diseases was extraordinarily high in community women who perceive this intervention as a useful and motivational strategy to increase awareness and adopt better behaviors. The mobile health intervention was highly accepted by participants who responded in the quality interviews. Unfortunately, a notable number of participants were lost during the follow-up due to external circumstances reported in informal discussions. Weak internet connectivity, user mistrust, lack of training has been reported in previous studies as barriers in the adoption of a mobile intervention for the prevention and control DZC [18]. However, many participants in this study continued to being a part in the large project. Women showed a particular interest and willingness for participating in the application of the new insecticidal coating which was also reported in the FDGs and chat reviews. In terms of usability, this study showed high proportion (86.6%) of users who read all messages and those (82.2%) who used it for responding and solving doubts. These findings are consistent with other studies which reported high acceptance and usability in mobile applications for the prevention and control of arboviral diseases [51–53] and other diseases [49,54]. Researchers have highlighted that WhatsApp, which is known by the population as a daily tool to interact with friends and relatives, allows a receptive space to exchange health information and solve doubts not commonly discussed during health care [50]. Despite high acceptance and good usability, some technical problems were presented, particularly with broadcast message reception, which need to be considered for further investigation.

The use of WhatsApp broadcasting is an affordable strategy that enables the specific targeting of groups such as community leaders, community volunteers, patients receiving home care, and DZC cases that require follow-up. This facilitates and maintains close cooperation and communication with the vector control or public health staff. Personnel cost can be avoided by task change in the communication departments of the MoH at central and local level. Although there is little evidence related to costs of digital technology in arbovirus management, according to a study, the costs associated with implementing a mobile application for disease prevention and surveillance can be influenced by factors such as community engagement, promotional activities, and one-time investments in technology [55]. The introduction of a mHealth program could also require an initial investment which should be considered in further research. It is important to note that privacy and data protection should always be incorporated. National laws should be reviewed, such as those in Colombia where regulations like Law 1581 of 2012 govern the protection of personal data [56]. Organizations planning to use WhatsApp for broadcasting should ensure compliance with these laws and regulations, which include obtaining consent from individuals before processing their personal data and implementing security measures to safeguard data.

### Suggestions for improvement of future studies

For the large intervention trial, WhatsApp can definitely be recommended for the prevention of DZC considering the following aspects:

- The close cooperation between local health authorities and other local associations is essential for increasing and sustaining the community participation as women could be multiplicators in their neighborhoods.
- Importantly, saving the WhatsApp contact in order to receive the broadcast messages was a condition in this study. It is important to supervise the registration process and mobilize friends and neighbors to remind each other. Vector control staff could also help during their routine inspection of household.
- It is recommended to directly save the participants’ contacts in cloud and do backup when recruiting. The creation of a broadcast list is facilitated thereby since manual adding of contacts into the phone and later into WhatsApp can be avoided. Gmail allows for data to be exported in vCard format to a phone’s sim card.
- WhatsApp business might be essential for a high number of participants and better chat management. For example, contact numbers should be placed in folders by area, and automated and timed messages should be set.
- Codes should be assigned to participants during the registration process for ensuring data protection.
- Motivation should be done creatively. People need to be reminded in a comfortable way of simple prevention steps, to not only follow commands of local health authorities but actually understand and self-apply vector control.
- WhatsApp should not be abused for the promotion of other purposes (such as commercials); messages should be short, clear and enjoyable.
- Staff cost can be avoided by task change in the communication departments of the MoH at central and local level.

### Potential limitations

This study had a limited observation period, possibility of ‘politeness bias’, and it is important to note that findings from the larger implementation trial are not included in this manuscript. While the absence of data from the larger trial may limit the depth of our analysis, it is worth mentioning that during the course of the trial, positive assessments and high compliance were observed among women. Furthermore, employing a mixed methods design enabled an analysis between the different pieces of the study, revealing high levels of agreement. This strengthens the reliability of our findings within the scope of this study.

Another limitation was the small sample size, which was not powered to evaluate behavior change. Authors recognize the significance of a more diverse sample and a control group for conducting a more insightful analysis. The analysis was focused on a sample representative of women, as they play a crucial role in vector control efforts at community level [40]. However, participants' loss exceeded our expectations (35% of women did not follow up on the qualitative analysis). This challenge was similar to another study that used WhatsApp messages for improving breast cancer knowledge in women [50]. Despite these challenges, the findings of this study allowed the assessment of the feasibility and relevant information concerning the implementation of a well-known mobile application for promoting preventative messages in arboviral diseases.

## Conclusion

This study introduced and tested the use of WhatsApp for the prevention of arboviral diseases demonstrating to be a feasible, acceptable, and affordable strategy. The content of messages was validated and tested by community women as part of a large intervention trial which is being conducted in an urban community of Colombia. In general, the results obtained suggest that WhatsApp could be incorporated as an additional tool in traditional health communication actions and vector operations for combating DZC.

## Supplementary Material

Supplemental Material

## Data Availability

The authors confirm that the data supporting the findings of this study are available within the article or its supplementary materials [additional file 2,3].

## List of abbreviations

mHealth: mobile health
DZC: dengue, Zika, chikungunya
IVM: Integrated vector Management
COMBI: Communication for Behavioural Impact
ICTs: information and communication technologies
SMS: short message services
Apps: applications
DANE: National Statistic Department of Colombia
ZMG: Center for Medicine and Society
IDS: the State Health Institute
MoH: Ministry of Health
KAP: knowledge, attitudes and practices
FGDs: focus group discussions
SPSS: the Statistical Packages for Social Sciences
CWP: changing the water in pet’s bowl
CLT: cleaning of laundry tank
VdR: Villa del Rosario
LP: Los Patios
