# Supplementary material for: WhatsApp-based intervention in urban Colombia to support the prevention of arboviral diseases: a feasibility study
Source: Pathog Glob Health. 2024 May 24;118(4):334–47. doi: 10.1080/20477724.2024.2358263 (PMC11238608; doi:10.1080/20477724.2024.2358263)
Supplement: Supplemental Material [file YPGH_A_2358263_SM7341.zip › Additional file 1 Messages.docx]

Description of the WhatsApp messages

| # of message | Whatsapp Broadcast Messages | Component | Purpose |
| --- | --- | --- | --- |
| 1 | Dengue, Zika and chikungunya are infectious diseases transmittable via “*Aedes*” mosquitos, which reproduce in clean water such as the water of your laundry tank. You can protect your family! | Health education | Spreading the type of water that mosquitos can reproduce in. Recognition of the word “*Aedes*” in vocabulary for distinction and success of large-scale campaigns |
| 2 | A breeding site can be any water container within and outside the house, where *Aedes* mosquitos lay their eggs, which develop into larvae, and finally into new mosquitos that may transmit dengue, Zika and chikungunya. | Health education | The first step to remove breeding sites is recognizing them. Increasing sensitivity for *Aedes* through repetition. |
| 3 | Empty or turn upside down any water container, such as buckets, pots, and bottles in order to avoid potential breeding sites for *Aedes* mosquitos in your house. | Behaviour change | Recognition of potential breeding sites.  Preventive practice: emptying and turning around water containers. |
| 4 | Do not forget to eliminate or throw away all unused containers that could accumulate water, for example old tires, toys. | Behaviour change | Recognition of potential breeding sites. Preventive practices: elimination of unused containers. |
| 5 | Allow the staff from the Departmental Health Institute entry your home and follow the instructions related to *Aedes* mosquito control to prevent dengue, Zika, and chikungunya. | Health communication | Cooperation and support for vector control staff. |
| 6 | Soon there will be a new method to fight the mosquitos in your house. It is a new transparent, protective coating, which is applied on the inner walls of your laundry tank. Lets participate! | Health promotion | Promotion of the coating for laundry tanks. |
| 7 | Change the water of your pet’s bowl and flowerpots at least every two days, to avoid that it turns into a breeding site for *Aedes* mosquitos. | Behaviour modification | Recognition of potential breeding sites and the frequency of changing water.  Preventative practice: Changing water of flowerpot and pet’s bowl. |
| 8 | Once a week clean your laundry tank with circular brushing to eliminate the mosquito’s eggs. Once your laundry tank is painted with the transparent, protective coating, a strong brushing will not be necessary. | Behaviour modification | Recognition of potential breeding sites and the frequency of cleaning.  Preventive practice: cleaning of laundry tank.  Instructions for applying the coating. |
| 9 | Did you already change the flowerpot and your pet’s water? Do not forget that you should do that every two days, in order to avoid a potential mosquito breeding site. | Behaviour modification | Promotion of behavioural change through repetitive reminders. |
| 10 | At least once a year, make sure that your elevated tank is well covered, so that mosquitos cannot enter. | Behaviour modification | Recognition of potential breeding sites.  Preventive practice: covering water containers |
| 11 | Very soon, your laundry tanks will be protected against *Aedes* mosquitos, if you apply the protective coating. After the application, you might see larvae, but they DO NOT develop into adult mosquitos. | Health promotion | Promotion and preparation for the large intervention trial.  Avoid false expectations. |
| 12 | Cut the wings of dengue! (local vector control staff prefer to use their slogan for consistency) | Health communication | Motivation and recognition of national campaign against dengue. |
| 13 | I hope you haven’t forgotten to clean your laundry tank, especially the walls, corners, and wall joints of your tank. Feel free to send us any message about your laundry tank. | Behaviour modification | Promotion of behavioural change through repetitive reminders. |
| 14 | Did you know that the female *Aedes* mosquitos are the ones that bite and transmit the virus of dengue, Zika and chikungunya? | Health education | Teach community; maintain attention through interesting detail. |
| 15 | Remember that children and elderly are the most vulnerable to be infected with dengue, Zika and chikungunya. Avoid breeding sites, which can be any container of clean water. Make sure to cover the buckets and tanks in your home or eliminate water containers that you are not using anymore. | Health education | Increasing awareness to protect vulnerable populations and remembering some preventatives practices |
| 16 | If you feel intense, continuing fever, if your bones, your head or your articulations hurt, if you lose your appetite and feel pain behind your eyes, go to your closest health center, it could be dengue! These are important symptoms of dengue! | Health education | Recognition at least 3 dengue symptoms for danger awareness. |
| 17 | Did you already cover or turn around all buckets, pots, and bottles in your house? If you do that, you will avoid that more female mosquitos can bite you. | Behaviour change | Promotion of behavioural change through repetitive reminders. |
| 18 | Remember that very soon a new protective coating will be applied to the tanks of the following areas: Santa Barbara, San Judas, Gran Colombia, La Palmita and El Paramo. If you have fish, you do not necessarily need to paint your laundry tank with this product. Fish are a great control method since they eat the mosquito’s eggs. | Health promotion | Clarification of the areas where the coating is to be applied and some instructions to avoid disappointment. |
| 19 | The coating will be easy to apply. Your laundry tank should be at least 24 h dry before starting to paint it. Paint only the inner walls (if there’s a roof that one as well), and finally let it dry for another 24h for the product to adhere in order to get a better result. | Health promotion | Preparation and instructions for the application. |
| 20 | The protective coating is very safe for people and for animals, therefore you can continue to use your laundry tank in the way you are used to. We hope that the protective coating will arrive to your house soon! | Health promotion | Increasing trust of population.  Promotion of the coating for laundry tanks. |
